# Characteristics of State Legislation Addressing Prescription Drug Price Increases in the United States, 2020

**DOI:** 10.1007/s11606-021-06838-x

**Published:** 2021-05-28

**Authors:** Arman A. Shahriar, Gabriela Vazquez Benitez, Pamala A. Pawloski, Steven P. Dehmer, Jonathan D. Alpern

**Affiliations:** 1 University of Minnesota Medical School, Minneapolis, MN USA; 2 HealthPartners Institute, Bloomington, MN USA; 3 Department of Medicine, University of Minnesota, Minneapolis, MN USA

## INTRODUCTION

Large price increases of prescription drugs are common in the USA.^1,2^ This practice creates affordability challenges for patients—particularly those with high-deductible plans and the uninsured.^3^ In response, states have developed legislation to address price increases, but little is known about legislation in this area. In this cross-sectional study, we provide a snapshot of current legislative activity by characterizing state price increase bills considered in 2020, in the context of price increase laws to date.

## METHODS

We searched the National Conference of State Legislatures (NCSL) prescription drug database under the topic “Pricing and Payment – Industry”, restricting the search to 2020 for bills and using the extent of the database (2015–2020) for laws—anticipating the earliest in 2017.^4^ We combined these results with legislative inventories and reports from the National Academy for State Health Policy (NASHP), a nonpartisan tracker of state drug pricing legislation, to generate our initial sample.

We included legislation incorporating ≥1 provision addressing drug price increases and excluded legislation that limited the scope of drugs (e.g., diabetes only or “15 costliest drugs”) because legislation limited to commonly prescribed or costly drugs may overlook drugs in smaller markets where price increases are common and substantial.^2,5,6^ We characterized 2020 bills by drug types targeted, price increase thresholds, reporting requirements, timing of reporting, sponsorship, and other characteristics. Data was collected 10/15/2020–01/15/2021.

## RESULTS

**Figure 1:**
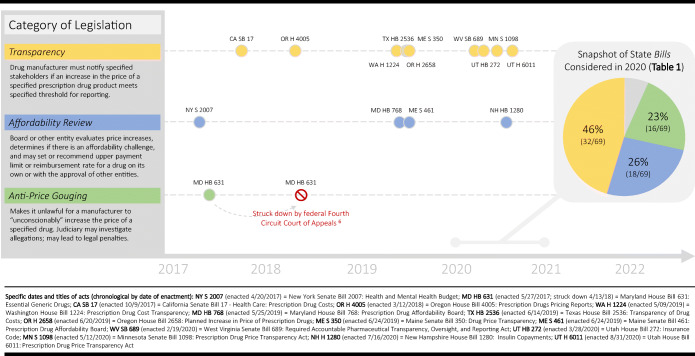
**Time line of** ***enacted*** **state prescription drug price increase legislation.**

After excluding 5 laws due to narrow scope, 15 laws (11 states) addressing price increases met inclusion criteria. These laws all fit within three categories (defined in Fig. 1): 10 transparency, 4 affordability review, and 1 anti-price gouging law in Maryland, later judged unconstitutional.^4^ After excluding 23 bills due to narrow scope (i.e., insulin; costliest/most commonly prescribed 10–25 drugs), 69 price increase bills were characterized. Most bills also fit within three categories: transparency (32/69; 46%), affordability review (18/69; 26%), or anti-price gouging (16/69; 23%). Aggregate characteristics of these categories are summarized in Table 1.

**Table 1:** Aggregate Characteristics of State Price Increase Bills Considered (Not Enacted) in 2020

	Transparency (32 bills)			Affordability Review (18 bills)			Anti-Price Gouging (16 bills)		
No. states	20 states			13 states			9 states		
		*n*	%		*n*	%		*n*	%
Drug types specified	No specification	25	78%	No specification	0	0%	No specification	3	19%
	Brand and generic	7	22%	Brand and generic	14	78%	Brand and generic	0	0%
	Brand, generic, and OPOE-brand	0	0%	Brand, generic, and OPOE-brand	4	22%	Brand, generic, and OPOE-brand	0	0%
	“Essential off-patent or generic”	0	0%	“Essential off-patent or generic”	0	0%	“Essential off-patent or generic”	9	56%
	All other (market shortage, “critical”)	0	0%	All other (market shortage, “critical”)	0	0%	All other (market shortage, “critical”)	4	25%
Objective threshold^a^	Yes (23 unique)	32	100%	Yes (9 unique)	18	100%	Yes (5 unique)	11	69%
	No	0	0%	No	0	0%	No	5	31%
Subjective threshold^b^	Yes	0	0%	Yes — price increase creates “affordability challenge” for patients or payors	18	100%	Yes — price increase is “unconscionable”, “unjustified”, or “unreasonable”	16	100%
	No	32	100%	no	0	0%	No	0	0%
Required reporting	yes — manufacturer notify variable entities^c^	32	100%	Yes — manufacturer notify commission/board	7	39%	Yes — manufacturer notify commissioner / board	1	6%
	No	0	0%	No — board to use “other means” (i.e. enter MOU with other states) to obtain pricing information	11	61%	No — other entities^d^ bring allegations of price gouging to the attorney general	15	94%
Timing of reporting^e^	Before price increase	18	56%	Before price increase	7	39%	Before price increase	1	6%
	After price increase	14	44%	After price increase	0	0%	After price increase	0	0%
	No manufacturer reporting required	0	0%	No manufacturer reporting required	11	63%	No manufacturer reporting required	15	94%
Other characteristics				Board has authority to set new reimbursement rate without approval of separate entity (e.g., legislative or judicial)	13	72%	Attorney general may investigate allegations; possible civil penalties	15	94%
				Board plan for new reimbursement rate must be approved by separate entity	3	17%	Superintendent may investigate allegations; possible civil penalties	1	6%
				Board authority unclear	2	11%			
Sponsorship^f^	Democratic party	11	34%	Democratic party	12	67%	Democratic party	13	81%
	Republican party	5	16%	Republican party	0	0%	Republican party	0	0%
	Bipartisan	16	50%	Bipartisan	6	33%	Bipartisan	3	19%

^a^Objective thresholds include a price threshold (e.g., a drug with a wholesale acquisition cost [WAC] of at least $40 for a 30-day course), a price increase threshold (e.g., a drug whose price increases 40% over 3 years), or both (e.g., a drug with a WAC of at least $100 for a 30-day course, whose price is increased by 20% over any 3-year period)

^b^Subjective thresholds, such as “unconscionable” price increases, do not specify prices or price increases and are open to interpretation by the specified entity (affordability board, judiciary, etc.)

^c^Entities needing to be notified by the drug manufacturers vary significantly in transparency legislation (11 total entities specified among 32 bills). Four most frequent: State health commissioner (6/32; 19%), Purchasers (6/32; 19%), State department of health (6/32; 19%), State department of insurance (5/32; 16%)

^d^Entities specified who may bring allegations to the attorney general include (but not limited to) the consumer drug protection commission, director of division of consumer affairs, health commissioner, health plans

^e^The category “after price increase” includes “by at least 30 days after the date of the increase”, “by at least 60 days after the date of the increase”, “quarterly”, and “annually”; the category “before price increase” includes “at least 30 days before the date of the planned increase” and “at least 60 days before the date of the planned increase”

^f^Bipartisan defined as at least 1 sponsor from each party, or a committee sponsorship

Note — one bill — MA HB 1133 / S 706 — is counted twice, both as “Transparency” and “Affordability Review” as the bill has elements of both

## DISCUSSION

Three categories of price increase legislation—transparency, affordability review, and anti-price gouging—account for all 15 state laws to date and 94% of bills considered in 2020. Most laws were enacted within the past 2 years, suggesting legislative momentum. As outstanding bills must be re-introduced in subsequent legislative biennia, our study offers lawmakers several considerations to enhance the likelihood that price increase legislation benefits patients.

Among the 32 transparency bills, 44% do not require manufacturer notice until after the price increase has occurred, possibly a response to legal pushback against California’s transparency law (SB17) requiring prospective notice.^4^ In theory, this post hoc stipulation compromises effectiveness, since patients may not become aware of a price increase until the point of purchase.

Maryland and Maine passed laws creating affordability review boards in 2019, followed by New Hampshire in 2020 (Fig. 1). Thirteen other states filed 18 similar bills in 2020. This legislation goes beyond transparency, allowing for payment limits when prices or price increases create “affordability challenges” for payors or patients.^7^ Unlike the Maryland and Maine laws—whose effectiveness has been questioned—most 2020 bills (72%) empower the board to set reimbursement levels for reviewed drugs without involving a separate entity.^7^ Additionally, many (61%) do not mandate manufacturer reporting, leaving procurement of pricing information to the board through “other means”, which may result in missed price hikes. Importantly, few (22%) specify off-patent off-exclusivity (OPOE) drugs—off-patent brand-name drugs without generic competition that are cheaper than patent-protected brand-name drugs but prone to price hikes.^2^ Failure to specify OPOE drugs subjects them to higher “brand-name” board review thresholds (e.g., increase of $3000 for a 1-year supply or “treatment course”) where smaller absolute price increases that are large on percentage basis may be overlooked.

Limitations include the exclusion of 23 price increase bills due to narrow scope, which are likely to lower state spending and benefit some patients, and the possibility of missing legislation enacted before 01/01/2015 or legislation missed by search criteria.

Modifications to legislative language relating to timing, reporting, and drug types could increase the impact of state transparency and affordability review legislation. Although no anti-price gouging laws have been enacted since Maryland’s, 2020 state lawmakers demonstrated a continued interest in this legislation. Recently announced model anti-price gouging legislation—including provisions to avoid repeating history—may be introduced as early as 2021.^8^
